# Harnessing Technology and Data to Unlock Actionable Insights in Implementation Research - Sleep Research Examples

**DOI:** 10.1093/geroni/igaf122.2060

**Published:** 2025-12-31

**Authors:** A Lynn Snow, Rosa Baier

## Abstract

Effective implementation of cutting-edge technology and data is dependent upon the development of solutions that are responsive to the unique needs of older adults and their environments. This symposium includes five presentations from the world of sleep research reflecting on efforts to develop and refine technology and data solutions for real-world implementation. The first and second presentations describe how interdisciplinary collaborations can result in new ways of bundling and using packages of multiple technologies to solve implementation complexities in the challenging arena of sleep environments. By using iterative pilot testing and multidisciplinary collaboration to inform technological development, the final technologies are much more acceptable and feasible than would be possible without collaborative iterative design. The third presentation reviews commercially available sleep tracking technologies, describes how technologies have evolved over time, and considers their implementation advantages and disadvantages. The final two presentations consider innovative ways of bringing data out of the analyst’s office and into the field so it can be dynamically used to inform and shape quality improvement and research in real time. The discussant, Dr. Rosa Baier, will reflect on the implications of these presentations for implementation research and quality improvement practice. Using the complexities of sleep research as a backdrop, this group of presentations offer an opportunity to consider how new insights can be stimulated through iterative field-informed packaging of technologies and data.

